# Adrenomedullin and its receptors are expressed in mouse pancreatic β-cells and suppresses insulin synthesis and secretion

**DOI:** 10.1371/journal.pone.0265890

**Published:** 2022-03-24

**Authors:** Yuanlin Dong, Simone Hernandez Ruano, Akansha Mishra, Kathleen A. Pennington, Chandrasekhar Yallampalli

**Affiliations:** Department of Obstetrics and Gynecology, Baylor College of Medicine/Texas Children’s Hospital, Houston, Texas, United States of America; Loma Linda University School of Medicine, UNITED STATES

## Abstract

Gestational diabetes mellitus (GDM) is associated with defective pancreatic β-cell adaptation in pregnancy, but the underlying mechanism remains obscure. Our previous studies demonstrated that GDM women display increased plasma adrenomedullin (ADM) levels, and non-obese GDM mice show decreased serum concentrations of insulin and the number of β-cells in pancreas islets. The aims of this study is to examine if ADM and its receptors are expressed in female mouse pancreas, and if so, whether insulin secretion is regulated by ADM in mouse β-cell line, NIT-1 cells and isolated mouse pancreatic islets. Present study shows that ADM and its receptor components CRLR, RAMPs are present in mouse pancreatic islets and co-localized with insulin. The expressions of ADM, CRLR and RAMP2 in islets from pregnant mice are reduced compared to that of non-pregnant mice. NIT-1-β cells express ADM and its receptor mRNA, and glucose dose-dependently stimulates expressions. Furthermore, ADM inhibits NIT-1-β cell growth, and this inhibition is reversed by ADM antagonist, ADM22-52. The glucose-induced insulin secretion was suppressed by ADM in NIT-1-β cells and isolated pancreatic islets from pregnant mice. These inhibitory effects are accompanied by upregulation of endoplasmic reticulum (ER) stress biomarker genes in NIT-1-β cells. This study unveils that reduced ADM and its receptors may play a role in β-cell adaptation during pregnancy, while increased plasma ADM in GDM may contribute to the β-cells dysfunction, and blockade of ADM may reverse β-cell insulin production.

## Introduction

During normal pregnancy, marked insulin resistance is accompanied by increased maternal insulin secretion [1]. This increased insulin resistance may contribute to facilitate the transportation of glucose and other nutrients toward the fetus. Maternal islets adapt to this increased demand mainly through increased β-cell proliferation and enhanced insulin secretion per β-cell [2]. Failure of these compensatory mechanisms may result in the development of glucose intolerance in pregnancy, known as gestational diabetes mellitus (GDM) in humans [3]. Clinical research has shown that GDM is associated with increased incidence of complications in both the mother and the fetus, including but not limited to maternal gestational hypertension and preeclampsia, and infant macrosomia and Caesarian delivery [4]. Furthermore, defective adaptation of the β-cells in the fetuses may be induced by the diabetic in-utero environment [5], resulting in the metabolic disorders in the offspring. Although efforts have been made in this area, the underlying mechanism for the defective adaptation of the β-cells are still unclear.

Adrenomedullin (ADM) is a potent vasodilator peptide with 52-amino acid residues originally isolated from human pheochromocytoma [6], and its action is mediated by calcitonin receptor-like receptor (CRLR). CRLR interacts with receptor activity-modifying proteins (RAMPs), among which RAMP2 and RAMP3 carry CRLR to the cellular membrane to confer high affinity for ADM [7]. ADM is expressed by a variety of cell types, and functions in an autocrine/paracrine manner [8], including cell proliferation [9], inflammation [6], hormone production [10], vascular adaptations in pregnancy [11], and fetal growth [12]. In humans, plasma ADM concentrations are elevated in obese individuals [13] and patients with T2DM type 2 diabetes [14]. Our previous studies have demonstrated that plasma ADM levels are increased in GDM women [15], and the pancreas β-cell number and serum insulin levels are reduced in a non-obese GDM mouse model [16]. These results may suggest an association between increased ADM levels and insulin insufficiency, but it is not clear if ADM plays a role in direct regulation of β-cell functions.

Reports show that rat pancreatic islets express ADM [17], and insulin-producing cells express ADM receptors [18]. We therefore suggest that ADM may play a role in the regulation of insulin secretion, and alterations in ADM concentration may regulate β-cell adaptation during pregnancy, and β-cell dysfunction maybe involved in the pathogenesis of diabetic pregnancy. Thus, the aims of this study were to determine if ADM and its receptors are present in non-pregnant and pregnant mouse pancreas, and if so, whether insulin synthesis and secretion by mouse pancreatic β-cell line and isolated pancreatic islets from pregnant mice were regulated by ADM. Here we show that ADM and its receptor components CRLR, RAMP2 and RAMP3 are present in both non-pregnant and pregnant mouse pancreatic islets and colocalized with insulin. The expressions of ADM, CRLR and RAMP2 in islets from pregnant mice are reduced compared to that of non-pregnant mice. Furthermore, ADM inhibits both mouse NIT-1 β-cells proliferation and insulin secretion, and these inhibitory effects can be reversed by ADM22-52. Finally, we demonstrate that ADM inhibits glucose stimulated insulin secretion in isolated pancreatic islets from pregnant mice, confirming thus confirming our cell line studies. Such evidence may implicate ADM as a fundamental factor in maintaining insulin homeostasis, and excessive ADM may be a possible causal agent in diabetic pregnancies.

## Materials and methods

### Animals

All animal procedures were approved by the Institutional Animal Care and Use Committee in Baylor College of Medicine and performed in accordance with NIH Guide for the Care and Use of Laboratory Animals. C57BL/6J female mice were purchased from Jackson Laboratory (Bar Harbor, ME, USA). Female mice at 9 weeks of age were housed with a male and checked daily for the presence of a vaginal plug (16). The day a vaginal plug was observed was designated as day 0.5 of pregnancy. Non-pregnant mice and the mice on day 13.5 and day 17.5 of pregnancy were sacrificed and the pancreatic tissues collected for either immunofluorescent staining with specific antibodies (n = 3/time point) or pancreatic islet isolation for glucose stimulated insulin secretion (GSIS) assays (n = 4 day 13.5 pregnant dams).

### Immunofluorescent imaging analysis

Paraffin embedded pancreatic tissues from non-pregnant and pregnant mice were cut at 5- to 7-μm thickness and mounted on Superfrost Plus Microscope Slides (Fisher Scientific, Pittsburg PA, USA). The sections were first deparaffinized and rehydrated, and then immunofluorescent stained. The α cells were detected by using goat anti-mouse glucagon antibody (1:200, Abcam, Cambridge MA, USA) followed by secondary antibody Alexa Flour 647 goat anti mouse IgG (1:200, magenta, Invitrogen, Eugene, Oregon, USA). The β cells were identified by using goat anti-guinea insulin antibody (1:100, Abcam, Cambridge MA, USA) followed by secondary antibody Alexa Flour 594 goat anti-guinea pig molecular probes (1:200, red, Invitrogen, Eugene, Oregon, USA). Furthermore, ADM and its receptor components were identified by using goat anti-rabbit ADM, CRLR, RAMP2, and RAMP3 antibodies respectively (1:50, Santa Cruz, Dallas TX, USA) followed by secondary antibody Alexa Flour 488 goat anti rabbit IgG (1:200, green, Invitrogen, Eugene, Oregon, USA) as previously described [19]. Negative controls were performed with only secondary antibody. The slides were then mounted with Mounting-medium containing 4′, 6-diamidino-2-phenylindole (DAPI; Vector Laboratories Inc., Burlingame, CA, USA) and viewed under an Olympus BX51 microscope and analyzed by using CellSence software (Olympus Scientific, Walthan MA, USA).

### Glucose Stimulated Insulin Secretion (GSIS) assays

Pancreatic islets were isolated from day 13.5 pregnant mice (n = 4) as previously described [20] and used routinely in our labs. Briefly, pancreas was infused with collagenase, removed and incubated in collagenase solution at 37°C. Once digestion was complete, samples were washed and ficoll gradient was applied to the isolated tissue. Cell pellets were resuspended in RPMI 1640. Islets were then picked, graded by size, and placed into culture overnight without or with ADM (10^-5^M). The following day islets were picked and placed in tubes with low (1.8 mM) or high glucose (16.8 mM) and with and without ADM. Media was collected following 30 min of culture to measure secreted insulin. Secreted and total insulin were measured by ELISA (Millipore) as previously described [16, 21, 22]. Percent secreted insulin was calculated and compared between groups to determine the effects of increased ADM on GSIS in islets from pregnant mice.

### Mouse NIT-1 β-cell culture

The mouse pancreatic β-cell line NIT-1 cells (ATCC CRL-2055, Manassas, VA, USA) were cultured in Ham’ F12K medium supplemented with 2mM L-glutamine (Corning Cellgro, Manassas, VA, USA), 1.5g/L sodium bicarbonate (SIGMA, St. Louis, MO, USA), 10% heat-inactivated dialyzed fetal bovine serum (GIBCO, Gaithersburg, MD, USA), 100 U/ml penicillin and 100 μg/ml streptomycin (Corning Cellgro, Manassas, VA, USA) in a 5% CO_2_ atmosphere at 37° C. NIT-1 cells (3 x 10 cells/well) were seeded in 6-well-plates and cultured for 3 days prior to the treatments. After a 30-min preincubation, the cells were treated with increasing doses of glucose (Sigma-Aldrich, St. Louis, MO, USA) in the presence or absence of ADM (10^−9^ M, American Peptide Co., Inc. Sunnyvale, CA, USA) for 24 hours. Total RNA was isolated using TRIzol (Life Technologies, Grand Island, NY, USA) and Reverse Transcription (RT) was performed for further Quantitative Real-time-PCR analysis.

### Quantitative Real-Time PCR

Quantitative Real-time PCR was performed using Taq universal SYBR Green Supermix (Bio-Rad, Hercules, CA, USA) [23]. PCR primers used for amplification of ADM (Mm.PT.58.11111908), CRLR (Mm.PT.58.10636953), RAMP2 (Mm.PT.58.30553776), and RAMP3 (Mm.PT.58.8586280) were purchased from Integrated DNA Technologies (IDT, Coralville, IW, USA), and the primer sequences for insulin and ER stress genes were list in Table 1. Amplification of both β-actin and GAPDH served as endogenous housekeeping controls. PCR conditions for SYBR Green gene expression were 10 min at 95°C for 1 cycle, then 15 sec at 94°C, 30 sec at 60°C and 15 sec at 72°C for 39 cycles. All experiments were performed in triplicate, and the average CT value of the two housekeeping genes was used to calculate the results using the 2–ΔΔCT method and expressed in fold increase/decrease of the gene of interest.

**Table 1:** Primer sequences.

	Forward	Reverse
*Insulin*	5’-GCTTCTTCTACACACCCATGTC-3’	5’-AGCACTGATCTACAATGCCAC-3’
*t-Xbp-1*	5’-TGGCCGGGTCTGCTGAGTCCG-3’	5’-GTCCATGGGAAGATGTTCTGG-3’
*s-Xbp-1*	5’-CTGAGTCCGAATCAGGTGCAG-3’	5’-GTCCATGGGAAGATGTTCTGG-3’
*Chop*	5’-CCACCACACCTGAAAGCAGAA-3’	5’-AGGTGAAAGGCAGGGACTCA-3’
*Bip*	5’-TTCAGCCAATTATCAGCAAACTCT-3’	5’-TTTTCTGATGTATCCTCTTCACCAGT-3’
*Β-actin*	5’-AGGTCATCACTATTGGCAACGA-3’	5’-CACTTCATGATGGAATTGAATGTAGTT-3’
*Gapdh*	5’-AGGTCGGTGTGAACGGATTTG-3’	5’-TGTAGACCATGTAGTTGAGGTCA-3’

### Cell proliferation assay

The cell proliferation assay was performed by using Cell proliferation Kit I (MTT) (Roche Diagnostics GmbH, Mannheim, Germany) according to the manufacture’s instruction. Briefly, NIT-1 cells were seeded in wells of 96-well plate containing various amounts of ADM (American Peptide Co., Inc. Sunnyvale, CA, USA) with or without ADM22-52 (American Peptide Co., Inc. Sunnyvale, CA, USA). After 72 hours preincubation, 10 μl of MTT labeling reagent was added and incubated for another 4 hours, followed by the treatment with solubilization solution for overnight. The absorbance at A550 were read and recorded by using Spectrophotometer CLARIO STAR (BMG Labtech, Inc., Cary, NC, USA).

### Insulin analysis

Insulin concentrations in NIT-1 cell culture medium were assessed using a Mouse insulin ELISA kit (Thermo Scientific, Frederick, MD, USA) according to manufacturer’s instructions. Briefly, NIT-1 cells were seeded in 96-well plates. After 3 days culture, the cells were treated with increasing doses of glucose (Sigma-Aldrich, St. Louis, MO, USA) in the presence or absence of ADM (10^−9^ M, American Peptide Co., Inc), and culture medium was collected 30-min and 24 hours after treatments. For insulin measurement, 100 μL of standards and culture medium were added into appropriate wells and incubate for 2 hours at room temperature, followed by incubation with biotinylated antibody, streptavidin-HRP solution and TMB substrate. The absorbance at A450 were read and recorded by using Spectrophotometer CLARIO STAR (BMG Labtech, Inc., Cary, NC, USA). The intra-assay coefficients of variation were <10% and intra-assay CVs were <12%.

### Statistics

All data were presented as mean ± SEM. Data were calculated and analyzed by GraphPad Prism (La Jolla, CA, USA). For comparison between 2 groups unpaired 2-tailed Student’s t test was used. For comparisons between 3 or more groups, a one-way ANOVA was used followed by a Bonferroni post hoc test was used for comparisons between groups. For analysis of mRNA expressions data, two-way repeated measures ANOVA was used, followed by Bonferroni post hoc analysis where appropriate. Statistical significance was defined as *p*<0.05.

## Results

### ADM and its receptor components are present in mouse pancreatic islets and co-localized with insulin

**Fig 1:**
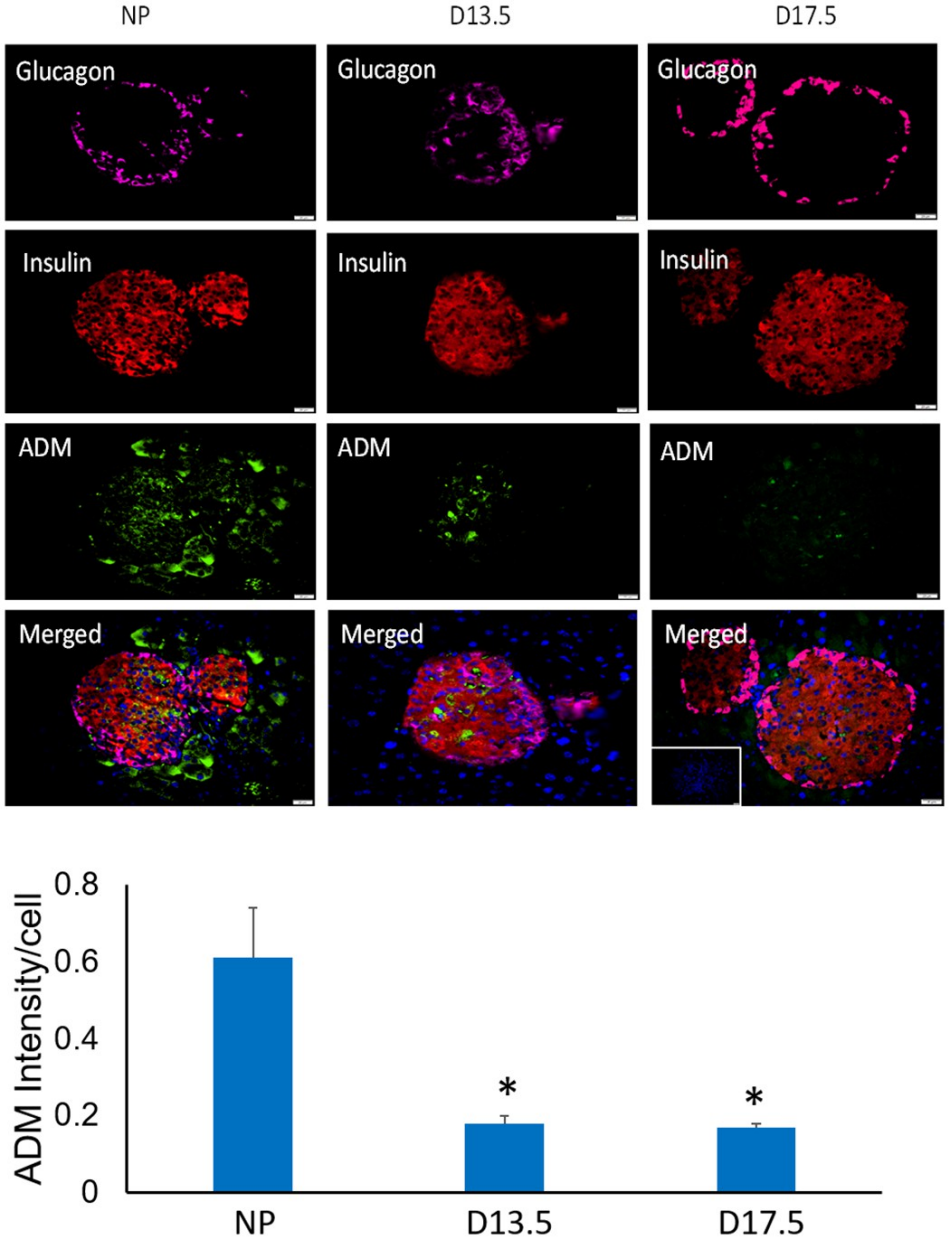
Cellular localization of ADM in mouse pancreas. Representative illustrative images showing triple immunofluorescence of glucagon (magenta), insulin (red), and ADM (green) in pancreas from nonpregnant (NP) and pregnant mouse on day 13.5 (D13.5) and day 17.5 (D17.5) of pregnancy. The merged column represents a composite of the above three images with cell nuclei (identified by DAPI, blue). Magnification, x 400. The labelings for ADM are generally restricted to the islets and colocalized with insulin. The data for ADM intensity per cell in the islets were displayed as mean+/-SEM (n = 4). * indicate significant difference when compared with NP (P<0.05). Inset is secondary only negative control.

**Fig 2:**
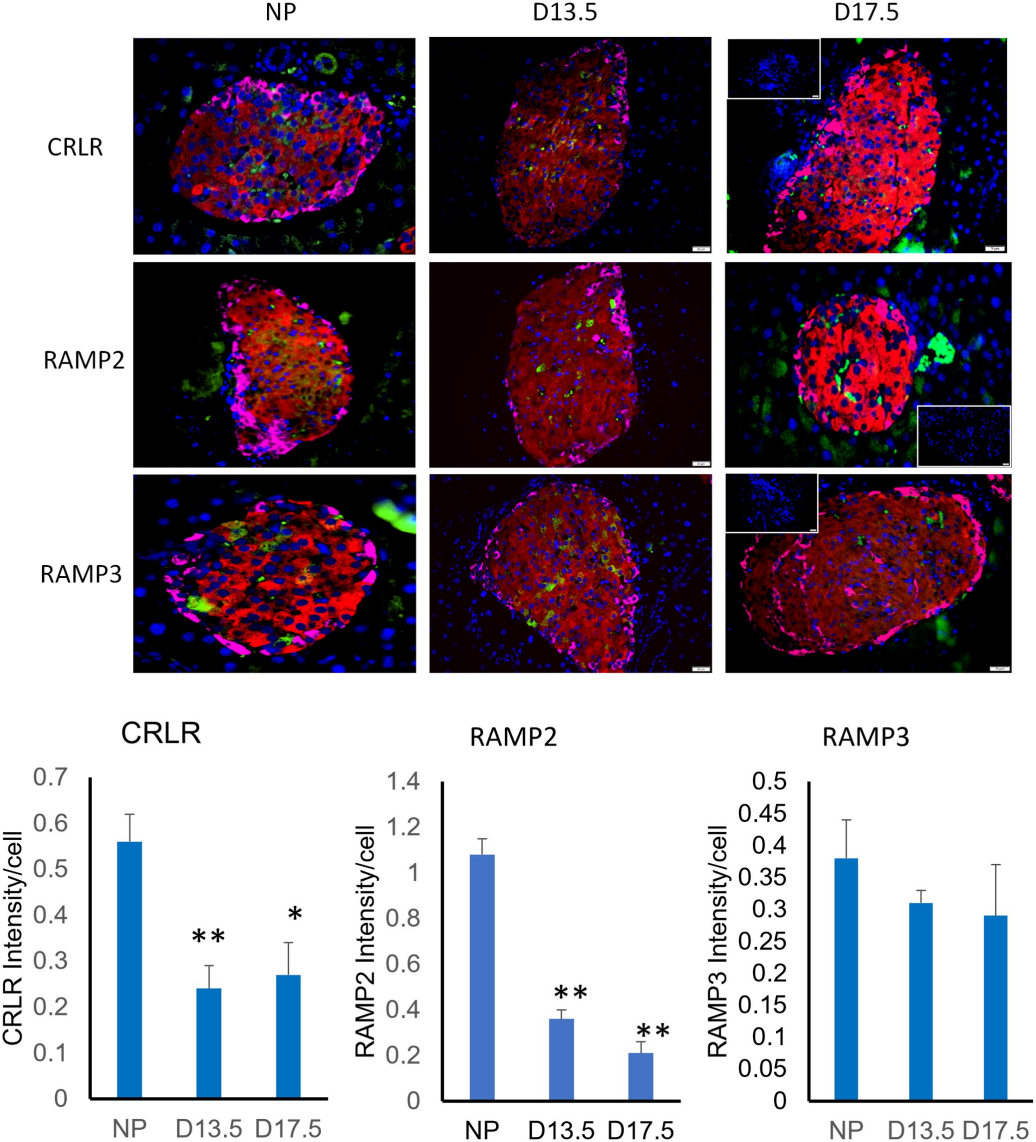
Cellular localization of ADM receptor components CRLR, RAMP2, and RAMP3 in mouse pancreas. Representative illustrative images represent a composite of the glucagon (magenta), insulin (red), and CRLR, RAMP2, RAMP3 (green) with cell nuclei (identified by DAPI, blue) in pancreas from nonpregnant mice (NP) and mice on day 13.5 (D13.5) and day 17.5 (D17.5) of pregnancy. Magnification, x 400. The labelings for CRLR, RAMP2 and RAMP3 are generally restricted to the islets and colocalized with insulin. The data for CRLR, RAMPs intensity per cell in the islets were displayed as mean+/-SEM (n = 4). * indicate significant difference compared with NP (P<0.05), and ** indicate P<0.01. Inset is secondary only negative control.

Triple immunofluorescence was applied to investigate the co-localization of the ADM and its receptor components with classical pancreatic hormones, glucagon and insulin, thereby identify the cell type in which these epitopes are expressed. In all cases, co-localization with insulin was observed for ADM, CRLR, RAMP2, and RAMP3 in the islets of pancreatic tissues from both non-pregnant and pregnant mice (Figs 1 and 2), whereas their immunoreactivity was rarely observed glucagon producing α cells, suggesting that a new endocrine function in the mouse pancreas, insulin-producing β-cells might be synthesizing and secreting ADM. Furthermore, the intensity of immunostaining for target proteins showed that no significant difference in the expressions of ADM and its receptor components was noted between animals on day 13.5 and day 17.5 of pregnancy (Figs 1 and 2), however, the expression for ADM, CRLR and RAMP2, but not RAMP3, are significantly reduced (p<0.05) in islets from pregnant mice when compared to that of non-pregnant mice, suggesting that alteration of ADM system in mouse pancreas may play a role in pregnancy-related β-cell adaptation.

### ADM blocks glucose stimulated insulin secretion in pancreatic islets isolated from pregnant mice

**Fig 3:**
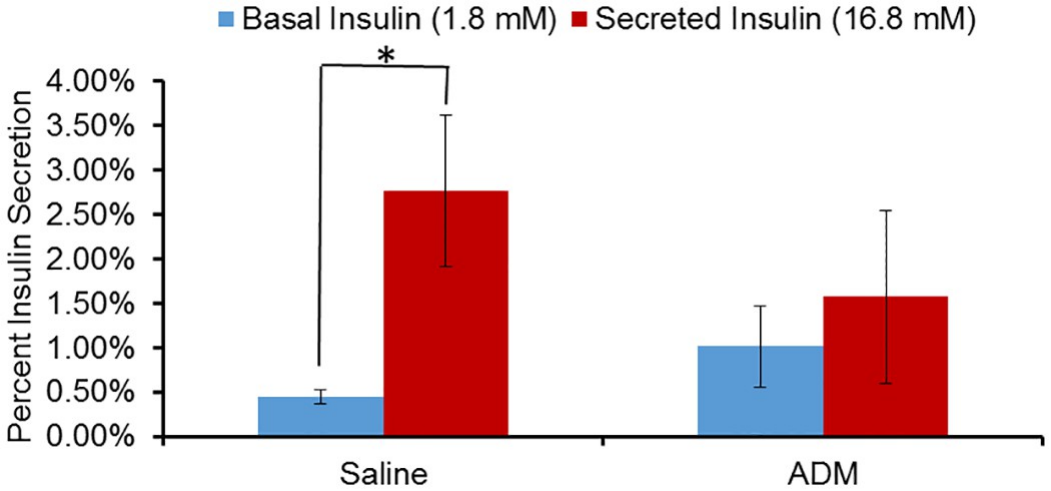
ADM inhibits glucose stimulated insulin secretion in pregnant mouse islets. Pancreatic islets were isolated from day 13.5 pregnant dams. GSIS assays were performed in the presence or absence of ADM. The data is displayed as percent secreted insulin +/- SEM (n = 4). * indicates significant difference between basal (in presence of 1.8 mM glucose) and secreted (in presence of 16.8 mM glucose) insulin.

Next, we assessed the effect of ADM on glucose stimulated insulin secretion using isolated pancreatic islets from day 13.5 pregnant mice. Islets cultured with ADM had inhibited GSIS while those cultured in saline had normal GSIS (Fig 3). These results indicate that increases in ADM levels can have negative effects on insulin secretion. To further evaluate the mechanisms by which ADM effects insulin secretion we transitioned to using the mouse β-cell line, NIT-1.

### NIT-1 β-cells express mRNA for ADM and its receptor components, and glucose stimulates the expressions for CRLR, RAMP2, and RAMP3

**Fig 4:**
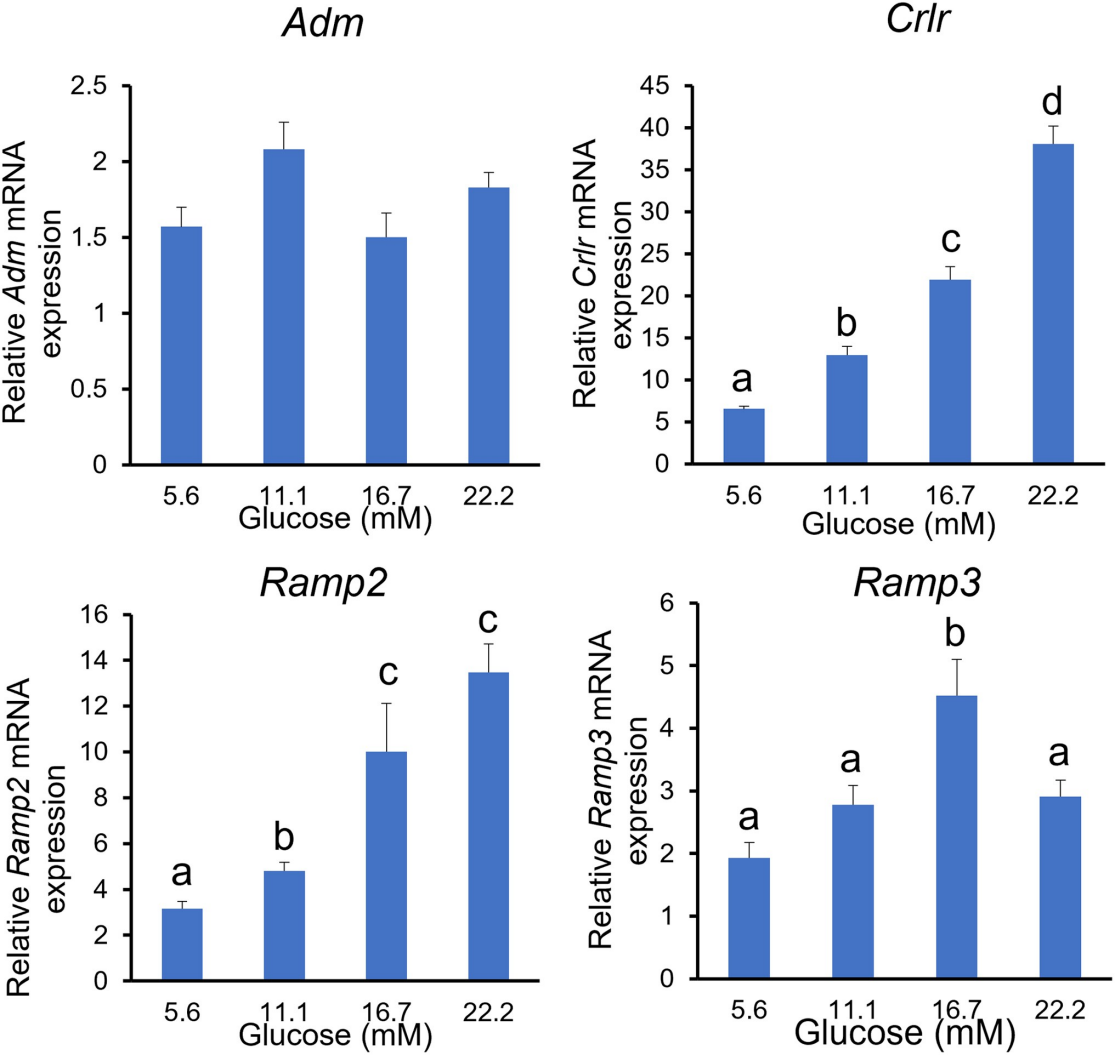
Glucose stimulates mRNA for ADM receptor components in mouse NIT-1 β cells. NIT-1 cells were incubated in Ham’s F12K medium with increasing doses of glucose (5.6–22.2 mM) for 24 hours, and the mRNA expression for ADM, CRLR, RAMP2, and RAMP3 were determined by using Real-time PCR with specific primers. The data were normalized to *β*-actin and GAPDH, and displayed as mean+/-SEM (n = 6). Different letters on each bar indicate significant difference between groups (P<0.05). Data showed that mRNA for CRLR, RAMP2, and RAMP3, but not ADM, are stimulated by glucose in a dose-dependent manner.

To determine the impact of hyperglycemia on ADM and its receptors in β-cells, we treated NIT-1 cells with increasing doses of glucose for 24 hours. As shown in Fig 4, increases in glucose concentrations from 5.6 mM to 22.2 mM resulted in a dose-dependent stimulation in mRNA expressions for CRLR, RAMP2 and RAMP3 (P<0.05), whereas the mRNA expressions for ADM were not significantly affected by the glucose treatment. These results indicated that increased glucose levels in GDM may contribute, at least in part, to the enhanced pancreatic β-cell ADM receptor expressions, thus enhancing ADM actions.

### ADM inhibits NIT-1 β-cell proliferation, and this inhibition is blocked by ADM antagonist

**Fig 5:**
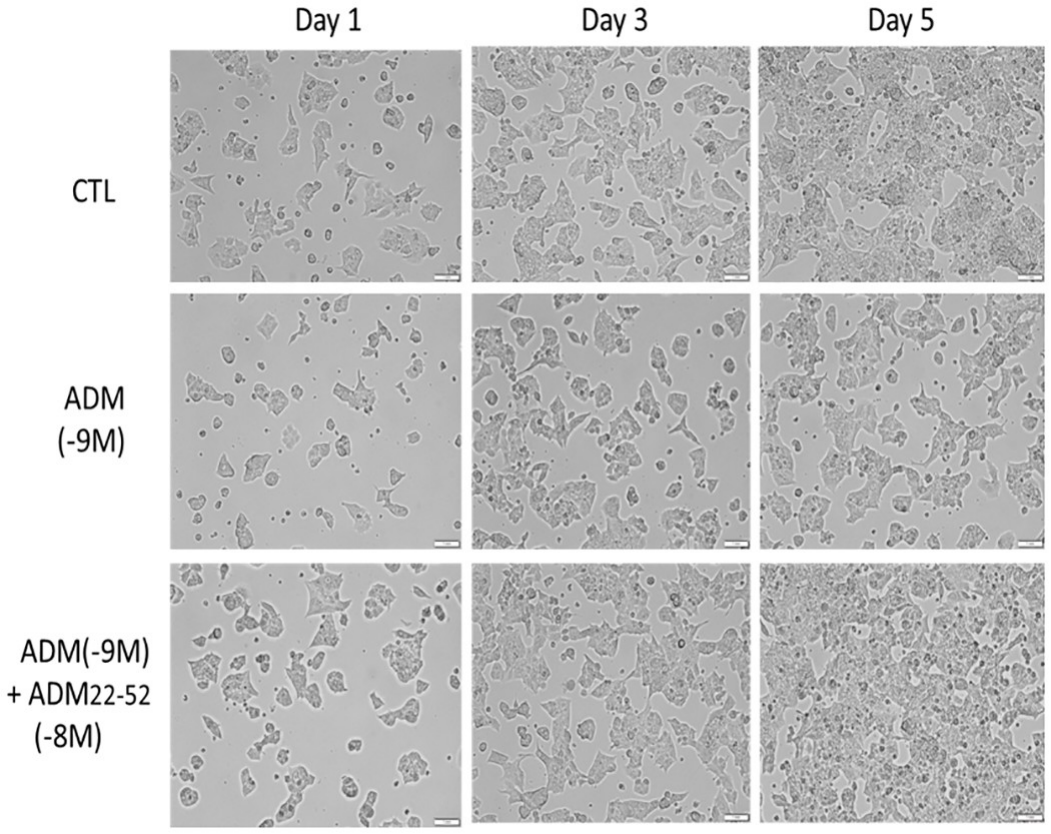
ADM inhibits NIT-1 β cell proliferation. Representative illustrative images showing the NIT-1 β cells grown in Ham’s F12K medium with 16.7 mM glucose and treated with ADM (10^−9^ M) in the presence or absence of ADM22-52 (10^−8^ M). The micrographs were taken on day 1, day 3, and day 5 after treatments. Compared with controls, profoundly decreased cell density was observed after 5 days ADM treatments, and this reduction was reversed by pretreatment of the cells with ADM22-52. Magnification, x 200.

**Fig 6:**
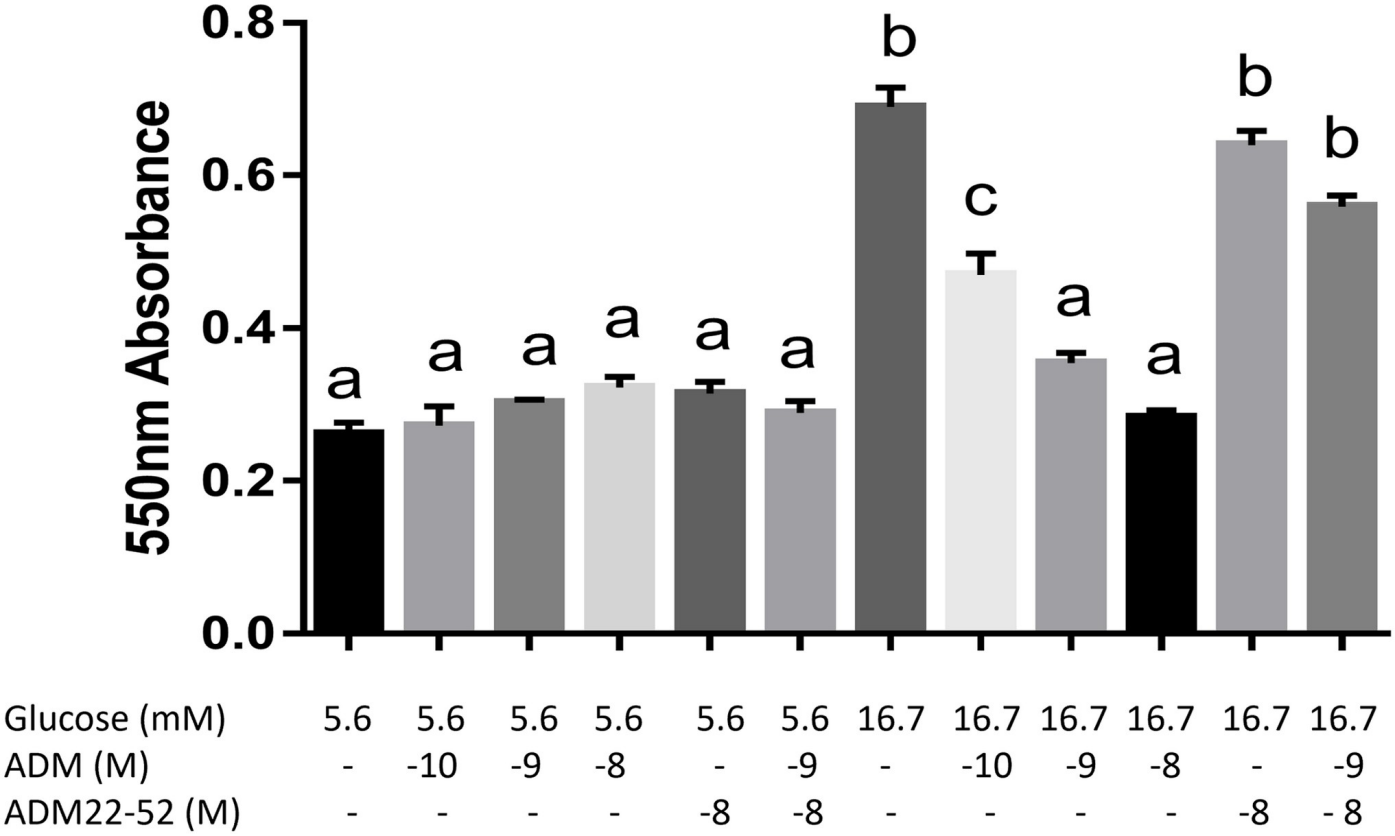
ADM inhibits glucose-stimulated NIT-1 β cell growth in MTT reduction test. NIT-1 β cells were cultured as described in the Materials and Methods. Reduction of MTT was colorimetrically determined after 72 hours treatments with ADM in the presence or absence of ADM22-52. ADM did not significantly affect the growth of NIT-1 cells in medium containing 5.6 mM glucose, but did significantly inhibit cell growth in medium containing 16.7 mM glucose, and this inhibition was reversed by ADM antagonist. The data displayed as mean+/-SEM (n = 4). Different letters on each bar indicate significant difference between groups (P<0.05).

To assess the influence of ADM on β cell growth, we treated NIT-1 cells in Ham’s F12K medium containing 16.7 mM glucose with ADM (10^−9^ M) in the presence or absence of ADM22-52 (10^−8^ M) for up to 5 days (Fig 5). The micrographs showed a clear reduction in cell density after 5 days of ADM treatment, and this decrease was reversed by pretreatment of the cells with ADM22-52. Furthermore, the MTT reduction test demonstrated that ADM did not affect the cell proliferation in medium containing 5.6 mM glucose (Fig 6), but did inhibit cell proliferation in medium containing 16.7 mM glucose in a dose-dependent manner, and this inhibition was reversed by ADM22-52, confirming that the inhibitory action of ADM on NIT-1 cell proliferation is mediated through ADM receptors.

### ADM inhibits glucose-induced insulin mRNA, synthesis and secretion by NIT-1 β-cells

**Fig 7:**
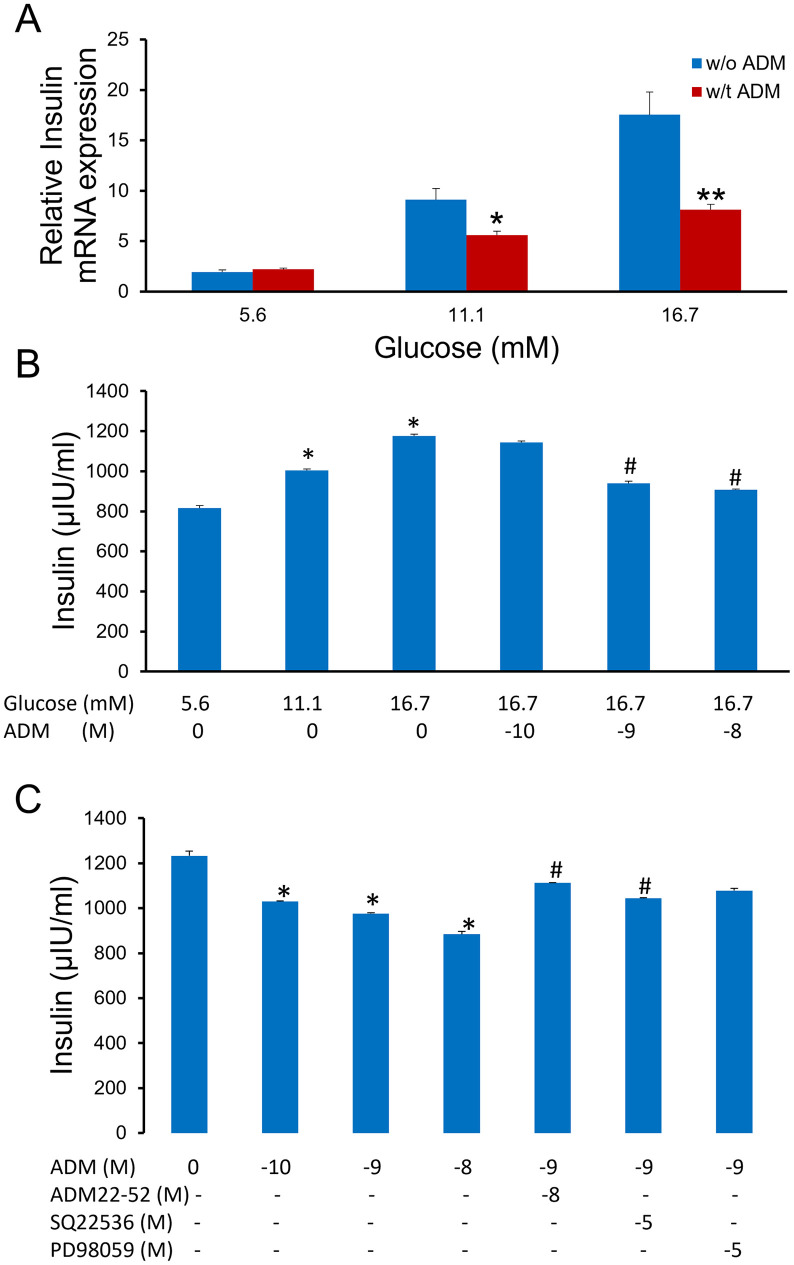
ADM inhibits glucose-stimulated insulin mRNA expression, secretion and synthesis in NIT-1 β-cells. (A) NIT-1 β-cells were incubated in Ham’s F12K medium containing increasing doses of glucose (5.6–16.7mM) with or without ADM (10^−9^ M) for 24 hours (n = 6). Real-time PCR was performed to determine the mRNA expression of insulin by NIT-1 cells. mRNA was normalized to housekeeping genes of *β*-actin and GAPDH. (B) NIT-1 β-cells were grown in medium containing increasing doses of glucose (5.6–16.7mM) with or without ADM for 30-min, after which insulin concentration in the medium was measured using ELISA kit. (C) NIT-1 β-cells were grown in medium containing 16.7mM glucose and ADM in the presence or absence of ADM22-52, SQ22536, and PD98059 for 24 hours, after which insulin concentration in the medium was measured using ELISA kit. Data displayed as mean+/-SEM (n = 3). * and # indicate significant difference vs. paired controls (P<0.05).

To determine the effects of ADM on insulin mRNA expressions by NIT-1 cells, we treated the cells with increasing doses of glucose in the presence or absence of ADM (10^−9^ M) for 24 hours (Fig 7A). The results showed that glucose stimulates NIT-1 cell insulin mRNA expression in a dose-dependent manner, whereas ADM inhibits this expression starting from 11.1 mM of glucose incubation. In addition, raising glucose from 5.6 to 16.7 mM led to an approximately 150% increase in insulin concentration after 30-min glucose stimulation (Fig 7B), and the addition of ADM to NIT-1 cells resulted in a dose-dependent reduction of insulin secretion. This inhibition reached 75% for an ADM concentration of 10^-8^M. In addition, to determine the underlying mechanisms of ADM actions, NIT-1 cells were pretreated with ADM antagonist ADM22-52, adenylyl cyclase inhibitor SQ22536, and Erk pathway inhibitor P98059 prior to the addition of ADM (Fig 7C). The results showed that ADM dose-dependently inhibits insulin synthesis in the cell culture medium after 24 hours treatments. The inhibitory action of ADM on insulin synthesis was reduced by ADM22-52, SQ22536, and PD98059, implying that ADM receptors, adenylyl cyclase, and Erk pathway are involved in the ADM inhibitory actions.

### ADM upregulates ER stress biomarker genes

**Fig 8:**
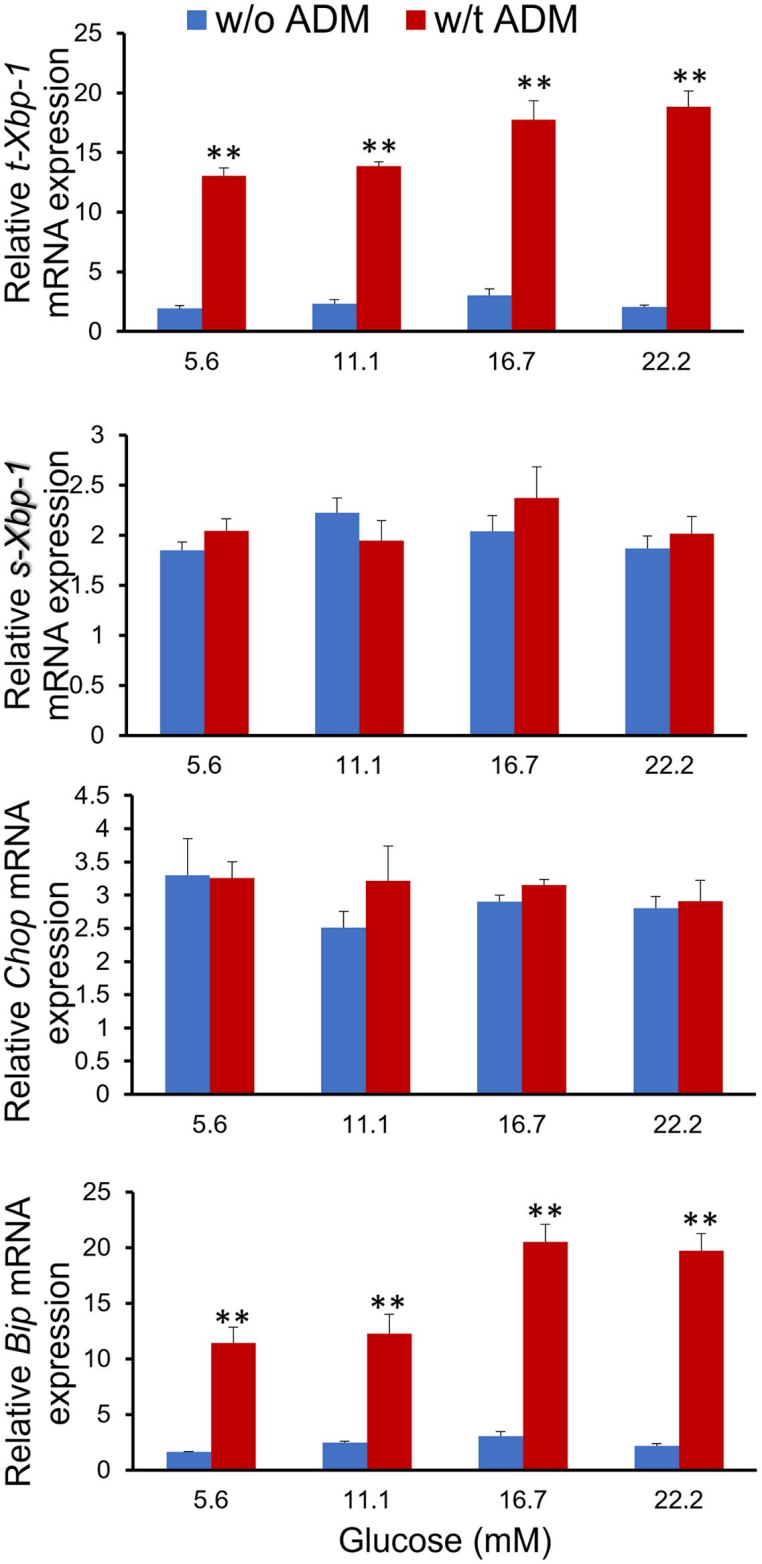
ADM induces ER stress in NIT-1 β cells. NIT-1 cells were cultured in Ham’s F12K medium containing increasing doses of glucose with or without ADM (10^−9^ M) for 24 hours. mRNA expressions for ER stress markers t-Xbp-1, s-Xbp-1, Chop, and BIP were determined by Real-time PCR, and normalized to housekeeping genes of β-actin and GAPDH. The data displayed as mean+/-SEM (n = 6). ** indicate significant difference between ADM treated and control groups (P<0.01).

Next, we assess the impact of raising glucose and ADM treatment on ER stress in NIT-1 β cells. NIT-1 cells were incubated in culture medium containing 5.6 to 22.2 mM of glucose in the presence or absence of ADM (10^−9^ M) for 24 hours, and ER stress markers were determined by using Real-time PCR. As shown in Fig 8, incubation of NIT-1 cells with increasing dose of glucose does not significantly affect the expression of t-Xbp-1, s-Xbp-1, Chop, and BIP, suggesting that these ER stress makers were not stimulated and Xbp-1 splicing was not activated by increasing glucose treatment. In contrast, addition of the ADM (10^−9^ M) in the culture medium led to an approximately 9-fold increase in mRNA for t-Xbp-1 compared to glucose-matched controls (P<0.01). Further, ADM significantly stimulated BIP mRNA expression, and this stimulation reached up to 10-fold higher than glucose-matched controls (P<0.01). These results suggest that ADM but not raising glucose causes activation of ER stress, and that is not accompanied by Xbp-1 splicing.

## Discussion

GDM is characterized by reduced adaptation of the pancreatic β-cells to the increased demands during pregnancy [24], but the molecular basis for these alterations remains unclear. The present study utilized pancreatic tissues from non-pregnant and pregnant mice, insulin producing β-cell line, and isolated mouse pancreatic islets to demonstrate that: 1) ADM and CRLR, RAMP2 and RAMP3 are abundantly expressed in mouse pancreatic islets and co-localized with insulin. 2) ADM block glucose stimulated insulin secretion in mouse pancreatic islets. 3) The expressions of ADM, CRLR and RAMP2 in islets from pregnant mice are lower compared to that of non-pregnant mice. 4) NIT-1 β-cells express mRNA for ADM and CRLR, RAMP 2, and RAMP3, and the expressions for receptor components, but not ADM, are stimulated by glucose in a dose-dependent manner. 5) ADM inhibits NIT-1 cell proliferation, and this inhibition is reversed by ADM antagonist, ADM22-52. 6) ADM inhibits NIT-1 cells glucose-induced insulin synthesis and secretion 7) these inhibitory effects were reversed by ADM22-52, adenylyl cyclase inhibitor, SQ22536, and Erk pathway inhibitor PD98059 in NIT-1 cells, and 78) Treatment of NIT-1 cells with ADM resulted in upregulation of ER stress biomarker genes, BIP and t-Xbp-1, indicating the involvement of ER stress in ADM’s action. We therefore propose that ADM maybe involved in the regulation of β-cell functions in an autocrine and paracrine manner. Reduced ADM and its receptors expression during pregnancy may play a role in promoting β-cell mass and insulin production, while increased plasma ADM in GDM may contribute to the β-cells dysfunction, and blockade of ADM may reverse β-cell insulin production.

### The cellular localization of ADM system in pregnant mouse pancreas

Four major cell types, α, β, δ and PP cells are found in the endocrine pancreas, and secrete hormones into the bloodstream [25]. The α cells produce glucagon and β cells produce insulin, to maintain glucose homeostasis. The δ cells produce somatostatin and PP cells produce pancreatic polypeptide that modulate the secretory function of the other cell types. It has been reported that ADM appears at day 11.5 in rat development coinciding in pancreas with glucagon and insulin in the same cells [26]. While at some time in rat development, all the cell type express ADM and then progressively evolve towards the adult pattern, where only the PP cells display a strong immunoreactivity for ADM. Meanwhile, the mRNA for ADM and its receptors have been shown to exist in six different insulin-producing cell lines, including RINm, N289, TR4, CRL2057, CRL1777, and CRL2055 [17], suggesting that the expression of ADM and its receptors in endocrine pancreatic cells seems to be a highly conserved feature from both the phylogenetic [27] and the ontogenetic [26] perspective. In the present study, we demonstrated that ADM and its receptor components CRLR, RAMP2, and RAMP3 are expressed in the pancreatic islets of non-pregnant and pregnant mice and co-localized with insulin (Figs 1 and 2). This finding was confirmed by immunofluorescence in the pancreatic tissues and by molecular analysis of mRNA expression in an insulin-producing mouse NIT-1 β-cell line. The pattern of distribution and endocrine cell type in the mouse are in an agreement with those previously reported by other groups [17, 28]. The co-localization of ADM and its receptors in the pancreas implicates that ADM may play a role in the control of both normal and altered pancreatic physiologies. Further, the expressions for ADM, CRLR and RAMP2, but not RAMP3, are significant reduced in islets from pregnant mice when compared to that of non-pregnant mice, this may imply that alteration of ADM system in mouse pancreas may play a role in pregnancy-related β-cell adaptation. In addition, our results in mouse NIT-1 cell line showed that glucose dose-dependently increased mRNA expressions of CRLR, RAMP2 and RAMP3, but not ADM per se (Fig 4). These findings provide new insight into the association between hyperglycemia and enhanced ADM action in diabetic pregnancies, indicating that glucose intolerance in GDM may be one of the stimulants for the enhanced ADM influence through increased ADM receptor on β-cells, thus contributing to the impaired insulin secretion.

### ADM inhibits β-Cell growth and glucose-stimulated insulin secretion

Conflicting results have been reported regarding the effect of ADM on insulin secretion. ADM has been reported to stimulate [29] or inhibit [17] insulin secretion in isolated rat islets. We report here that ADM inhibited glucose stimulated insulin secretion in pancreatic islets isolated from pregnant mice, further confirming previous findings in rat [17] and corroborating our previously published work using a human β-cell line [15]. However, isolated pancreatic islets used in these studies consist of various cell types and thus may not be appropriate in evaluating the impacts of ADM on β-cell functions as well as ADM mechanisms of action. Therefore, in this present study, we utilized a differentiated mouse insulinoma cell line NIT-1 to determine the direct effect of ADM on β-cell growth and insulin secretion and synthesis and investigate the mechanisms of ADM action on β-cell growth and function. This NIT-1 cell line retains various differentiated features of native β-cells [30] and is considered a suitable model for studying the β-cell proliferation, insulin secretion and biosynthesis [31]. Here, we show that ADM did not affect the cell proliferation in medium with 5.6 mM glucose, but did inhibit cell proliferation in medium with 16.7mM glucose in a dose-dependent manner (Figs 4 and 5), and this inhibition was blocked by pretreatment of cells with ADM22-52, indicating that enhanced ADM inhibitory action in hyperglycemia may result from glucose-stimulated ADM receptor overexpression, and the ADM inhibitory effect are mediated through ADM receptors. In addition, our results showed that glucose stimulates NIT-1 cell insulin mRNA expression as well as insulin secretion in a dose-dependent manner (Fig 6A and 6B), and ADM inhibits both glucose-stimulated insulin mRNA expression and secretion. Furthermore, ADM dose-dependently inhibits insulin synthesis in the cell culture medium after 24 hours treatments (Fig 6C). Our results are consistent with the effects of ADM on β-cells described by other groups in rat models. Martinez et al reported that a monoclonal antibody against ADM increases insulin release 5-fold more than controls in isolated rat islets [17]. Sekine et al showed that pancreatic beta cells ADM exposure resulted in a reduction in insulin secretion [32]. ADM administration in diabetic SHR/ N-cp rats in vivo decreases serum insulin levels with a concomitant increase in circulating glucose levels [33]. Such experimental evidence may implicate ADM as a fundamental factor in maintaining insulin homeostasis and normoglycemia, and dysregulation of ADM maybe one of the causal factors in diabetes. Collectively, further investigation focused on the development of blocking agents for ADM may result in new treatments for pancreatic ADM-related disorders.

### Underlying mechanisms of ADM actions

Glucose stimulates insulin secretion by generating metabolic coupling factors which promote β- cell exocytosis of insulin granules [34]. In physiological circumstances, mitochondrial metabolism of glucose increases ATP levels and leads to closure of the ATP-sensitive K+ channel, thereby resulting in the β- cell membrane depolarization. The opening of the voltage-gated calcium currents (VGCC) then induces Ca2+ influx into the β-cell and promoting insulin exocytosis. The mechanisms of ADM inhibitory actions on β- cell functions may include the regulation of glucose metabolism that is related to the β-cell excitability, the membrane potential or the VGCC, intracellular cAMP concentration, and the exocytosis machinery of insulin granules. In the present study, we observed that the inhibitory action of ADM on insulin synthesis was abolished by ADM22-52, SQ22536, and PD98059 (Fig 6C). The effects of ADM22-52 suggest that the effects seen were due to ADM-ADM receptor interactions and not a non-specific toxic effect of ADM on β-cells. The finding that SQ22536 reverses ADM inhibited insulin release supports the notion that cAMP is the second messenger for the ADM actions [7], and confirm that exocytosis of insulin granules is stimulated through the elevation of cAMP [35]. PD98059 blocks ADM actions, indicating that the members of the mitogen-activated protein kinase family, extracellular response kinase-1/2 (Erk1/2), are activated in the β-cell after ADM addition [36]. Therefore, a better understanding of the ADM system in pancreatic physiology and pathological states, such as GDM, may help define new areas of therapeutic intervention to improve the metabolic homeostasis.

### ADM induces ER stress in β-cells

Physiologically required for the folding, export, and processing of newly synthesized insulin, pancreatic beta cells possess a highly developed ER [37]. Served as an ER chaperone and a sensor of protein misfolding, immunoglobulin heavy chain binding protein (BIP) plays a central role in this process [38]. ER stress developed and apoptosis is induced by enhanced transcription of C/EBP homologous protein (CHOP) [39, 40], and X-box binding protein 1 (Xbp-1) is activated when misfolded proteins are accumulated in the ER [41]. Increasing evidence indicates that ER stress may be directly involved in the β-cell dysfunction and death observed during the development and progression of diabetes mellitus [42], and overwhelming ER stress can also induce oxidative stress [43], which may further impair β-cell function. In the present study, our Real time-PCR analysis showed significant upregulation of ER stress genes t-Xbp-1 and BIP (Fig 7), suggesting that ADM is capable of activating ER stress and dysregulating the unfolded protein response (UPR) pathway, which inevitably leads to marked β-cell dysfunction. We also showed decreased β-cell growth and proliferation after exposure to ADM (Figs 4 and 5), further confirming ER stress-mediated apoptosis. Therefore, we propose from these data that ADM induces excessive activation of ER stress and subsequent failure of the UPR, may contribute to eventual β-cell dysfunction.

Further studies are required to examine if administration of ADM to pregnant mice could induce GDM symptoms with impaired β-cell functions, whether reduced number of β-cells in pancreas islets and lowered circulating insulin concentration in non-obese GDM mice are associated with increased ADM and its receptor expression in pancreatic islets, and if administration of ADM antagonist could mitigate impaired insulin production and glucose intolerance in vivo. Nonetheless, our current study provides the evidence that ADM could be involved in both the physiological regulation of insulin secretion in normal pregnancy and the pathogenesis of diabetic pregnancy as a causative factor of impaired insulin synthesis and secretion, and blockade of ADM actions in GDM patients with its antagonists may improve β-cell functions.

## Supporting information

S1 Dataset (XLSX)
